# The Source and Credibility of Colorectal Cancer Information on Twitter: Erratum

**DOI:** 10.1097/01.md.0000482827.02221.a5

**Published:** 2016-05-06

In the article “The Source and Credibility of Colorectal Cancer Information on Twitter”,^1^ which appeared in Volume 95, Issue 7 of *Medicine*, Figures 1 and 2 were originally mislabeled. The article has since been corrected online.
